# Medical and legal aspects of telemedicine in ophthalmology

**Published:** 2019

**Authors:** Gianfranco Gioia, Mauro Salducci

**Affiliations:** * Faculty of Medicine and Dentistry, Department of Sense Organs, Master in Medical Legal Ophthalmology, Sapienza University of Rome, Italy

**Keywords:** telemedicine, ophthalmology, ethics, data protection

## Abstract

Telemedicine provides adequate medical assistance for physically distant patients by using the Information and Communication Technologies (ICT). Telemedicine includes techniques and tools for health monitoring and care implemented through systems providing rapid access to both specialists and patients. Telemedicine may link human and economic resources. Telemedicine may facilitate the access of patients to specialized healthcare in places lacking qualified personnel or in remote or difficult access areas thus reducing long waiting lists and high costs for the health systems. Telemedicine projects between different countries are developing, but ethical and legal issues are emerging. This article refers to telemedicine as a broad concept of distance medicine. The main purpose will be the medical-legal aspects. We will also describe the telemedicine in ophthalmology and the main issues raised by its implementation.

## Telemedicine 

Telemedicine provides welfare services by using the ICT when the health professional and the patient are distant. Telemedicine allow the reliable transmission of medical information (texts, sounds, images, etc.) for the prevention, diagnosis, treatment and monitoring of patients [**1**]. Telemedicine is useful for doctors and patients, particularly in rural areas or in developing countries without good health infrastructures [**2**]. It can play an important role in the development of health care all over the world. Telemedicine is helpful in remote group collaboration between health professionals from different countries because it consists of real time communication between the participants. In this way, professionals in distant positions can communicate with experts on a particular issue. This behavior is part of good medical practice [**3**-**6**]. Moreover, the patients seeking a second opinion from distant doctors may use telemedicine. Telemedicine may also be part of preventive medicine by giving patients information on their health conditions and monitoring chronic diseases such as diabetic retinopathy, glaucoma, macular age-related degeneration, etc. By means of telemedicine, we do telemonitoring of patients [**7**] thus avoiding long waiting lists and reducing hospitalizations. The electronic prescription in telemedicine avoids errors, such as mistakes in the tax code, the patient’s age, or the dosage, consequently to incomprehensible calligraphy of the doctor [**8**]. The improvement of healthcare is the real goal of telemedicine. Notwithstanding all the advantages, there is no telemedicine in Europe. The difficulties of application are the costs of telemedicine services, the difficult technical interoperability across countries, the lack of defined ethical standards and regulations [**9**].

## Telemedicine and ophthalmology

Telemedicine has multiple applications in ophthalmology because it needs visual images for the diagnosis, therapy, and follow-up of the diseases. Ophthalmology has quickly adapted to the cybernetic world and uses the telemedicine technologic system to access advanced healthcare. Its effective use has shown economic and health benefits [**10**,**11**]. For example, telemedicine may improve the quality of health care in two important ophthalmological diseases, such as diabetic retinopathy (DR) and retinopathy of prematurity (ROP). DR is a cause of blindness among working age adults. In particular, the telemonitoring of DR is helpful for clinicians. Telemedicine programs provide screening for DR patients in primary assistance [**12**,**13**]. Consequently, the spreading of the ophthalmological programs will promote the access to retinal screening or eye tests with the appropriate frequency [**14**]. Image-based diagnosis and telemedicine are reliable, accurate, and cost-effective for ROP in premature babies [**15**,**16**]. The imaging technology in telemedicine is good for the documentation of the retinal fundus thus also improving ophthalmologic care. Telemedicine in ophthalmology is useful for intraocular pressure monitoring and macular disease monitoring. Finally, remote consultations and examinations, instead of face-to-face, are gaining ground thanks to two-way communication technologies in emerging clinical institutions [**17**].

## The legal framework 

According to the articles 56 and 57 of the Treaty on the Functioning of the European Union (TFEU), telemedicine is a free service. According to the the European law, telemedicine is a health service and an information service (an electronical service provided for a fee at a distance). Therefore, both the regulations relating to health care and the regulations relating to information society services are applicable [**18**].

As far as information and telecommunication are concerned, we need to consider the following documents: Directive 95/46/European Union (EU) [**19**], General Data Protection Regulation (GDPR) [**20**], Directive 98/34/EU, Information Society Services Directive [**21**,**22**], Directive 2000/31/EU, Directive on electronic commerce [**23**], Directive 2002/58/EU and Directive on privacy and electronic communication [**24**]. Directive 2011/24/EU, the so-called cross-border directive, regulates health services [**25**]. The EU has increased the presence of telemedicine in Europe, as a standard medical service for European patients protected by the social security system [**26**]. The EU’s efforts are also quite economically significant (over € 500 million in research funding on eHealth instruments). However, the value of telemedicine does not hide its complex difficulties. For example, when health workers from different countries collaborate, there is no consensus on language to use for data recording in the patient’s medical record or who is responsible for technical training (the so-called e-literacy) of health professionals. All electronic health records (EHRs) need to integrate into a global eHealth infrastructure. Despite several working documents, we lack a set of rules on telemedicine in Europe. Most of the regulatory positions still depend on Member States with the best skills in health care. The approach to telemedicine varies in European countries. Even at national level, legal voids threaten the practice of telemedicine, leaving unprotected patients and health workers [**27**].

## GDPR: privacy in health

Published in the European Official Journal on May 4 2016, it entered into force on May 24 2016, but its implementation took place after two years, therefore starting from May 25 2018. Regulation 2016/679 represents the European legislation about data protection. Its purpose is the definitive harmonization of the regulation on the protection of personal data. With the Lisbon treaty, the protection of personal data is a fundamental right in EU. By increasing citizens’ trust in the digital society thanks to the more stringent protection, the regulation is functional to the digital development of the EU and safeguards the free movement of personal data. With the European regulation, we move from a proprietary vision of the datum (not treatable without consent) to a vision of control of the data enhancing the free circulation. There is the fundamental right of patients to the confidentiality of their medical records. Patient privacy is undeniable unless it is revoked (i.e. informed, non-coercive) when it neutralizes the public interest. The information disclosed should be limited to such information or part of the medical record. Telemedicine creates problems consequent to the involvement of non-clinical staff in teleconsultations, and the vulnerability of transmission lines to security breaches. Healthcare professionals and public health organizations carry out the processing of health data according to a specific discipline [**20**]. The confidentiality needs to guarantee for the health data of citizens in health facilities. Health data about intimate details of the patients are subject to a general prohibition of dissemination, as well as enhanced protection (article 4 of GDPR) [**20**]. Health data is all that personal information suitable to reveal a person's state of health and body and mental conditions. Genetic data and photographs taken for surgical interventions or controls are also health data. Furthermore, as established by the Court of Cassation (Civil Cassation, United Sections, judgment 12.27.2017, number 30981), sensitive data suitable to reveal the state of health must be treated with organizational methods to protect them, such as encryption, thus making the data subject unidentifiable. According to the European regulation health data can be used only for purposes related to health (treatment purposes), for the supervision of the National Health System and for research in the public interest. These purposes allow the possibility to introduce special conditions or additional limits for treatment. In this sense, the Italian legislator has provided, with the new Privacy Code, further measures to protect health data set by the national control authority and reviewed every two years. The article 9 (letter h) of the GDPR does not consent data processing for purposes of preventive medicine or occupational medicine, such as the assessment of the employee's ability to work, diagnosis in accordance with Union or Member State law or health professional contracts. However, the rule allows the Member States to introduce limitations with regard to the processing of genetic data, biometric data, or health data. The Italian legislator, with the decree to update the Privacy Code, has introduced the possibility that the Guarantor imposes specific guarantee measures (additional to the normal security measures) for the treatment of health data [**28**]. The directive 680 of 2016 is similar to the GDPR. Many of the rules contained are equivalent to those of the new European regulation on privacy and personal data. They have a specific scope of application and concern the processing carried out by the competent authorities for prevention, investigation, public security, etc. This directive expresses the need to serve the data for the time to achieve the purposes and to verify the need for storage or deletion after deadline. The appointment of the data protection officer is also obligatory for the judicial authority because of the assistance that this figure can provide in the complex treatment of sensitive data. The text provides administrative sanctions against violations concerning the methods of processing and introduces penal sanctions for illegitimate purposes in the treatment [**24**].

## The informed consent

Before proceeding with data collection, it is necessary to inform the patient. The document about informed consent indicates the subject collecting data, the purposes of the treatment, the methods of treatment, the subjects to whom the data can be communicated, the identification details of the holder and the methods to protect your data. The Italian Ophthalmological Society has elaborated the informed consent forms. The informed consent is the tool through which information about the intervention is disposable for the patient in order to gather his consent to proceed. Therefore, it presupposes two distinct moments: information and the actual consent. The informed consent consists of the specific information sheet of the intervention about the execution procedure, the post-treatment, advantages and complications. The act of consent is the same for all the information sheets [**29**]. The article 1, according to the principles set out in articles 2, 13 and 32 of Law 219/2017 of the Constitution, states that no medical treatment starts or continues without the informed consent of the interested person, except in cases expressly provided by law [**30**], such as the treatment for the public interest. The Italian legal system establishes the freedom of choice of the care place (article 32 of the Constitution and Legislative Decree 502/92) and the freedom to undergo treatment or not (article 1 of Law 219/2017). According to the GDPR, once citizens have decided to undergo a treatment they cannot refuse consent to the processing of data for treatment and diagnosis purposes. The digital preservation process aims to make a document usable over time, in its "digital essence" and in accordance with the new privacy regulation (European Regulation 679 on protection of personal data) [**20**]. The Digital Administration Code asserts that the Information Technology (IT) document and its processing recognize the equivalence between a computerized and paper document. Decree of the President of the Council of Ministers of the Italian Republic (DPCM) of 13 November 2014 sets the technical rules for the IT document. DPCM of 3 December 2013 preceded it, published in the Official Gazette number 59 of 12 March 2014, and related to managing IT documents and their preservation. The technical rules on preservation follow international [**31**] and national standards [**32**].

## The Electronic Sanitary File (ESF)

The ESF, according to the article 12 of Legislative Decree 179/2012, is an IT tool that combines data and documents (digital or digitized) of health and social health types related to the patients. Its function is to share such data and the patient's medical history among various doctors or health organizations. Authorized healthcare personnel and the patient (with safe modes, e.g. smart card) can access the ESF. Since the article 12 of Decree Law 179/2012 is active, the inclusion of data within the ESF depends on the consent of the patient (article 3 bis). In this regard, you know about who has access to your data and how these data are used. The patient has the right to revoke the consent and to obscure some specific data from the ESF. Although mobile electronic medical records are beneficial, several factors contribute to their not full potential use. Training in functionality and reliable infrastructures might foster tablet implementation [**33**]. The European Union of Medical Specialists (EUMS) has published a document that contains the "European Definition of Medical Act". This definition, adopted for the first time in 2005, was later amended in 2006 and finally in the Brussels meeting of 25 April 2009. This is the approved version: "The medical act covers all professional activities, for example of scientific, teaching, training, educational, organizational, clinical and medical technology, carried out in order to promote health, prevent diseases, make diagnoses and prescribe therapeutic treatments or rehabilitative treatments for patients, individuals, groups or communities, in the framework of ethical and deontological rules. The medical act is a responsibility of the qualified physician; it must be performed by the doctor or under his direct supervision and/ or prescription". Patients disclosing personal information to their physicians need to protect the same information against incidents to the treatment process. In this sense, there is no distinction between traditional medicine and telemedicine. The duty of confidentiality applies to all information provided to a tele-consulting doctor by a third party such as a healthcare professional [**34**].

## The information disclosure consent 

Most patients understand that the primary physician responsible for their case will need to ensure that all team members have the information to fulfill their professional roles in the care process. It is necessary to inform the patients about disclosure of information about them to others involved in their health care. If the doctor decides to divulge confidential information, he must be ready to explain and justify his decision. Because of the need to inform members of the support team, the law assumes the patient's implicit consent to disclose information to them. A final point concerns the circumstances in which the patient's refusal to disclose can be canceled by the doctor or other authorities. These circumstances arise when medical conditions of a patient pose a serious threat to the community in general. Health status data are visible to third parties, such as relatives, family members, voluntary personnel, after the patient, if conscious, has been informed and allowed. In any case, it is necessary to respect the request of the person hospitalized to secret his presence in the health facility or information on his health conditions even to the legitimate third parties [**35**]. The issue of the access right to the patient's medical record obviously binds to the more general right of access to administrative documents (governed by the article 22 and following Law 241/1990) and to the treatment of personal health data according to the code about personal data protection referred to legislative decree number 196/2003. The public medical record is subject to the productivity of incident effects on subjective legal situations of public relevance (see Criminal Court, Section V, 21 November 2011, Number 42917). The prevailing doctrine attributes juridical relevance to the medical record, as it is the only instrument capable of processing patient care information to allow communication between the various health care workers. The doctor, in charge of compilation, is a public official pursuant to article 357 of penal code, while the person responsible for the storage and good keeping is always the head of the department where the patient is undergoing treatment. The doctor is required a particular accuracy in writing all elements in the medical record: diagnosis, therapy, outcomes, etc., according to article 24 of the Decree of the Minister of Health of 5 August 1977 and arranged according to the procedures set forth in article 26 of the Code of Medical Deontology. The medical record together with the related reports need indefinite preservation, as it is an official act essential to ensure the certainty of clinical data, as well as being a valuable source of documentation for research of historical health data. In this panorama, the question of limits on access to third parties to the information contained in the medical record belonging to another subject has legal importance [**36**].

## The Network and Information Security (NIS) directive for health data protection

Several devices are useful for remote communication, such as video conferencing unit, e-mail, webcam, smartphone, etc. The channel to allow communication is variable (broadband, network, wireless). The legal perspective of confidentiality focuses on the relationship between the subjects involved rather than on the systems by which they communicate, but we must pay attention to this last aspect. We consider the NIS directive as the first step in the European cybersecurity strategy. Approved by the EU Parliament on 6 July 2016, the directive aims to reinforce the security and IT resilience within the Old Continent. The need of NIS directive starts from a consideration: networks, systems, and information services play a vital role in today society. Without them the internal market could not work, so their reliability and security are essential for economic and social activities. The NIS applies to two categories: the operators of essential services that are necessary to the maintenance of basic social and economic activities (such as health, transport and energy companies), and the digital service operators such as search engines, e-commerce platforms, etc. Both of them need to adopt appropriate technical and organizational measures to manage risks and prevent IT incidents. In the event of default, very severe penalties will be present, ranging from a minimum of 12 thousand up to 150 thousand euros. In this regard, it is necessary, that at national level, an intervention group for cyber security and a national authority responsible for the security of networks and information systems are designated in the event of an accident. Furthermore, the NIS establishes the cooperation group composed of representatives of the Member States, the Commission and the European Network and Information Security Agency (ENISA), a team to promote collaboration between the countries of the Union in relation to the security of networks and information systems in order to facilitate the information exchange. The two regulations, i.e. the GDPR and the NIS directive, overlap when an IT security incident involves violation of personal data [**37**].

## Electronic Identification Authentication and trust Services (eIDAS)

The eIDAS is the European regulation governing electronic signatures, money transfers and other types of electronic transactions in the European single market. It has allowed the creation of standards for electronic signatures, digital certificates and other forms of electronic authentication, thus allowing for the replacement of paper documents with digital equivalents that have the same legal value and official recognition in all EU countries. The member countries of the EU are required to recognize electronic signatures that meet the standards set by eIDAS. In particular, it distinguishes three types of signatures inherent sensitive data. The director adheres with local, state, federal, and international guidelines for the acquisition of health information, transmission, and storage of data by interfacing with electronic medical records and widely available archiving and communication systems, preferably using standards-based interoperability protocols. Imaging performance and diagnostic acquisition devices in ophthalmology should follow periodic manufacturer's recommendations. Diagnostic displays need of periodic checking and recalibration for normal function. The security, integrity, and availability of data (including backup and archiving) are the tasks of the IT staff. The setting, i.e. a medical office, urgent care, emergency, or community health care center, should be able to monitor the vital parameters of the test, to make a detailed medical history review, to conduct and transmit telemedicine for ophthalmological examination and to organize appropriate follow-up and care. Telemedicine exams will have different space requirements based on their use of synchronous technology or asynchronous technology. Synchronous visits require space for the patient, the local provider, and the remote provider to conduct the exam in private and discuss the results. Synchronous visits typically require audio and video equipment, a computer to transmit the necessary information about the exam and the devices and technologies to conduct a remote eye examination. Asynchronous visits require space for the necessary imaging equipment, other devices used and space for the preparation and presentation of images and data. Generally, a small space within an existing clinical space is adequate for telemedicine examinations and consultations. There are few normative indications concerning the personnel who acquire, transmit, and interpret the data of telemedicine. Each member of the team has the necessary qualifications established by the program. An activity-based (or "function-based") assessment of staff requirements is necessary. The reading method for image analysis should be transparent to the applicant. The reading center is responsible for reading errors. The basic requirements for staff involved in ophthalmology telemedicine in a remote imaging site are diagnostic equipment, the awareness of the risks and the clinical estimation of ocular complications due to pupillary dilatation (if imaging is under the mydriatic eyewash), universal precautions, antiseptic technique and informed consent. Training for safe and correct contact with patients in a clinical setting, as indicated by applicable hospital and facility standards, is necessary. Training on specific devices and equipment to obtain the necessary certifications and adherence to the quality control for the instruments is important. The ophthalmologists who work as distant doctors should receive initial training and periodic re-evaluation to meet quality standards for the activities performance. The doctor who reads the test must confirm the coverage of responsibility for medical negligence for this activity by the insurer. An expert reader, under the supervision of the physician, bases the current ophthalmology telemedicine on a store-and-forward model with image acquisition for subsequent evaluation. The most recent applications may involve home-based tests with transmission and web-based interactions by the patient [**37**].

## Network access, data transmission, Health Internet (Hi)-Ethics in telemedicine 

Telemedicine relies heavily on the video and audio transmission of data through telecommunication networks. Secure access to the network and data transmission is essential for the confidentiality of personal and medical data. The promise of this network is that patient information will be electronically available to authorized personnel wherever the patient is or information is necessary. The access needs at least one authentication check (password). The checks ensure that access is available only for authorized users. An appointed individual is responsible for the security of a connected system. All network workers are aware of their responsibilities. All the incidents threatening the security are under control. The most obvious way to reduce the risk of unauthorized access to computer data on the Internet is to control traffic through the interface between the local network and the external Internet. This is a function of a firewall. It is important to know that they cannot protect themselves from traffic that does not cross them. There are different types of firewalls such as the network-wide firewall using a router to make decisions, i.e. what to pass or block based on network protocols, usually Internet Protocol (IP) addresses, an application layer that is a system including a personal computer (PC) with two ports (one for entry and the other one for outgoing traffic). A firewall is a mean of ensuring that only the right traffic passes through. 

**Telemedicine includes four main areas:**

- live or synchronous audio-video telemedicine, i.e. bi-directional real-time communication between a patient and a healthcare provider using audiovisual telecommunication technologies and data collection; 

- store-and-forward or asynchronous telemedicine (freely translatable as "store and return"), i.e. electronic transmission of health data (images, text or other digital data) to a healthcare provider for the evaluation and provision of the service using methods other than real-time interaction with the patient; it is a technique in which information subdivided into packets in its path between the single stations (or nodes) of the network, must be totally received, before it can be retransmitted; 

- Remote patient monitoring (RPM), i.e. collection of health data directly from the patient, usually during the normal activities of daily life, transmitted to a healthcare provider for analysis and possible actions;

- Mobile Health: health care, patient communication and training based on mobile communication platforms, e.g. mobile phones, tablets, PCs, etc.

Encryption is therefore a powerful help to protect telemedicine transmission. There are two types of encryption algorithms. With secret key encryption, the sender and recipient both use the same key to lock and unlock the message. On the contrary, with public key cryptography each user has two unique keys, i.e. a public key and a private key. In private, the key is useful to encrypt any message sent as a digital signature. The recipient can decrypt the signature with the public key to verify the identity and authenticity of the message. The power of digital signatures is that they also detect very slight changes in a message. Informed users naturally expect that the clinical information on the Internet is high quality, accurate, timely, and evidence-based. Digital native users have less critical faculties. They are easily aware of the validity of what they read on the internet. Several organizations have tried to establish these principles in guidelines or codes of ethical practice for the construction of Internet sites. The Hi-Ethics consortium is a voluntary group aiming to join the websites and the most used health information providers whose goal is to gain consumer confidence in internet health services. The goals of Hi-Ethics are to offer internet services that reflect ethical and high quality standards, to provide reliable and up-to-date health information, to keep private and secure personal information and to take special precautions for personal health information. The Hi-Ethics allows consumers to distinguish online health services following these principles from those that do not [**38**].

## Telemedicine equipment and operational risks

There are two broad categories of equipment used in telemedicine for the ophthalmology program: information acquisition devices (camera, optical coherent tomography, tonometer, autorefractometer, campimeter, etc.), image communication devices (computers, servers, network devices, etc.), to send data. The remarkable technological development of biomedical equipment for therapeutic, rehabilitative, and diagnostic use has certainly brought great benefits, but it has created particular problems for the protection of personnel, patients, and health workers, in the health structures, mostly from electronic risks. Before using any biomedical equipment, make sure it has undergone regular acceptance testing and that the operators have received adequate and specific training on its correct use. For a long time, the CEI EN 60601-1 standard has been the reference for manufacturers and users of electro-medical devices for the diagnosis and treatment of patients. The international work of adaptation of the norm in these last years, after some modifications published in 1991 and 1995, has led to the publication of the third edition in 2006 in which the concept of safety has been expanded to include the aspect of the essential performance of electro medical equipment. The experience also extends to the legal aspects of telemedicine equipment. The basis of this legislative framework is the Consumer Protection Act of 1987 dealing with the general responsibility for artisanal products and applied to the teleconsultations equipment [**39**]. The regulations apply to Computed Tomography (CT) scanners, X-rays, ultrasounds, etc. All these devices (including new videoconferences and related equipment) must show the European conformity (CE) mark indicating that they comply with the appropriate safety, quality, and performance standards. The Medical Devices Agency (MDA) is also responsible for registering the manufacturer and reporting incidents, as well as for the general implementation and promotion of European directives. MDA has identified the repeated causes of adverse incidents with medical devices such as poor quality, outdated or worn out devices, incompatibility with auxiliary equipment, poor documentation, inappropriate use, inadequate training, maintenance errors, or lack of assistance. These comments mainly refer to malfunctions of the operational equipment [**40**]. Several devices are useful to communicate remotely, such as a video conferencing unit, e-mail, webcam, or smartphone. The channel allowing communication can be broadband, network, or wireless. The operational risks are those identified by the MDA as the main causes of adverse accidents. The analysis of these causes shows that they fall into two categories: inadequacies due to technology and those due to insufficient staff. We can distinguish four main technological risks involving: 

- the image quality: a patient has the right to expect that a consultant can draw the same correct conclusions from an image on a telemedicine screen as from a conventional face-to-face visit, particularly important for Ophthalmology surveys;

- the lack of suitable equipment for a health service;

- malfunctioning equipment: the breakdown of the computer or video equipment is unfortunately one of the most common features of telemedicine;

- inadequate guidelines: guideline are as a bridge between technology and participants in teleconsulting. The guidelines determine the teleconsultation process and the documentation provides an archive of therapy, prescriptions, dosages of drugs, plans, etc. This combination of protocol and recording of the action provides a powerful audit trail that can have considerable value in any legal dispute.

In Italy, the Directorate General for Medical Devices and the Pharmaceutical Service is responsible for the completion and implementation of the regulation of medical devices, including tasks related to market surveillance, accident monitoring, clinical investigations, and evaluation of technologies and address of Health Technology Assessment (HTA) activities [**41**]. As regards the operational risks due to the staff, the Commission refers to the definition of "healthcare professionals" as defined in the article 3/f of the Directive 2011/24/EU [**38**]. According to the Directive 2005/36/EC, healthcare professionals are the doctor, the nurse responsible for the general care, the dentist, the midwife, or the pharmacist. We have principles to ensure that each team member is aware of his responsibilities and those of the other members. The patient should also know who is responsible for his care. These are simple precautions to avoid complaints of negligence. Telemedicine has sensitized people on issues that underline legislative discrepancy about the problem of accreditation and qualifications of health staff to protect the patient from incompetent professionals [**41**,**42**].

## Discussion

The benefits of telemedicine are promising and, particularly, in ophthalmology that is a specialization based on diagnostic imaging. The application of telemedicine is useful for patients, for ophthalmologists and for health institutions as it allows access to medical care, it avoids unnecessary long movements, and it increases the profits by reaching patients, by expanding the spectrum of available services and by reducing health care costs and waiting lists. Telemedicine in ophthalmology allows collaboration between professionals from different locations, sometimes even from different countries. It simplifies, through different telecommunications channels and mobile technologies, direct patient access to ophthalmologists who practice in another city or country without requiring any of the participants to travel. However, some characteristics of telemedicine can become problematic, such as the violation of privacy, the physical distance, the inclusion of new technological methods, the purchase of expensive equipment, the weakening of the doctor/ patient relationship, the involuntary increase in opportunities for incorrect behavior, the delegation of functions due to the remote nature of the treatment process, etc. Health professionals should consider that telemedicine could lead them to face further potential medical errors within a substantially more challenging standard of care. The EU has not yet issued specific rules on medical liability, despite its specific features, and this gap can jeopardize the development of telemedicine in Europe thus denying all its benefits to European patients. However, the EU will not be able to create a uniform regulation covering all aspects of telemedicine, but it has already taken a step forward in terms of technology and privacy, which are in fact already well-defined topics under European law. Regarding the rights of patients in the field of telemedicine, the EU will be able to create some basic guidelines in the context of cross-border healthcare patients' rights. The rights of European patients still lack a set of uniform rules and the standardization in such a complex area as telemedicine perhaps it would not be the best choice. We cannot expect harmonized rules for the medical responsibility deriving from telemedicine. In fact, Member States have very different national laws to address these issues because the EU brings together models of continental law and common law models. Because of this intrinsic difference, the attempt to harmonize civil liability, especially criminal liability, will be doomed to fail. It would be the purpose for each Member State to provide a legal framework for telemedicine, while the role of the EU would be limited to requiring Member States to regulate it. When telemedicine takes place across national borders, or in Europe, there is likely to be even more opportunity for legal discord on which laws to apply, i.e. the laws of the country where the telepatient is present or of the country where the teleconsultation took place. What happens if telemedicine is legal to practice in one country but is not in another? We need to conform a telemedicine task force in ophthalmology involving the academic and research world by using excellent resources, and collaboration between the Italian Ophthalmology Society, the Italian Association of Doctors Ophthalmologists and the Society of Legal Medicine in Ophthalmology to establish the legislation and guidelines of the telemedicine. The health manager or health director of telemedicine is necessary to avoid the delegation of care to less qualified subordinates and to establish their competences and responsibilities. Actually, telemedicine is not a defined discipline to justify the professional accreditation of "Ophthalmologist Specialist in Telemedicine". Tele-education or eLearning should become a standardized process with certification requirement as well as continuous courses are necessary to spread the current European legislation in telemedicine both at the Ministry of Health and Medical Orders levels in all Italian regions. In conclusion, it is a good investment of efforts and resources because the worldwide future of telemedicine in ophthalmology undoubtedly promises well.
